# Trends of Empiric Antibiotic Usage in a Secondary Care Hospital, Karachi, Pakistan

**DOI:** 10.1155/2013/832857

**Published:** 2013-12-25

**Authors:** Syed Rehan Ali, Shakeel Ahmed, Heeramani Lohana

**Affiliations:** Department of Paediatrics & Child Health, Aga Khan University Hospital, Karachi 74800, Pakistan

## Abstract

*Objectives*. (1) To determine the indications, frequency, and types of antibiotics used in hospitalized paediatric patients at tertiary care hospital and (2) to evaluate whether the prescribed antibiotics were based on the isolation of organism and their sensitivity. *Study Design*. Descriptive observational hospital based study. *Results*. A total of 131 patients were included over 6 months of study period, in whom antibiotics were prescribed at the time of admission. The majority were between 1 and 5 years of age. M : F ratio was 1 : 1. Fever was the commonest symptom (in 84% of cases) followed by gastroenteritis. Blood culture was done in 114 cases (87%) and was positive only in 10 (8.8%). The commonest organism isolated from blood was *Salmonella* Typhi. Ceftriaxone was found to be the most frequently prescribed antibiotic as an empirical therapy. 102 (77.86%) patients received Ceftriaxone, followed by ampicillin. The antibiotics were probably used on the basis of clinical condition rather than the result of blood culture, as yield of blood culture was quite low. *Conclusion*. Our study showed an unjustified use of antibiotics regardless of the admission and discharge diagnosis in acute febrile illnesses. Further on, inappropriate practice of using Ceftriaxone was noted in LRTI and pneumonia. Efforts are needed to educate physicians about the rational use of antibiotics.

## 1. Introduction

Antimicrobial agents are among the most frequently prescribed drugs. Inappropriate use of these agents is associated with allergic reactions, toxicity, super infection, and more importantly development of antimicrobial resistance [1]. The excessive and inappropriate use of antibiotics adds an unnecessary economic burden to health care system and coincides with increase in drug resistant organisms [2]. It has been observed in many studies that patients with drug resistant organisms require longer hospitalization and had increased risk of mortality [2]. Rational drug therapy is defined as the use of drugs only when there is specific need. Once the need has been established, then a proper drug has to be selected on the basis of efficacy, safety, cost, effectiveness, availability, acceptability, and dosage form [3]. On the other hand, inappropriate or irrational use of drugs is described by James Trostle as “consumption of drugs in a way that reduces or negates their efficacy or in a situation where they are unlikely to have desired effect” [3]. A study conducted at Karachi showed improper use of antibiotics in acute watery diarrhea in 39% of cases [4, 5]. Various studies have highlighted the problem of inappropriate and irrational use of all groups of drugs in Pakistan [6]. The aim of the study was to look at the indication, frequencys and types of antibiotics use in children admitted at a tertiary care hospital at Karachi and; whether if their use was rational based on isolation of microorganisms.

## 2. Materials and Methods

This is a hospital-based observational descriptive study conducted at the Department of Paediatrics and Child Health of the Aga Khan University Hospital, Karachi. The duration of study was 6 months; their study was conducted from 16 November 2010 to 15 May 2011. A Total of 131 patients were enrolled who satisfied the inclusion criteria.

Children between 2 months and 15 years of age were included in the study, in whom antibiotics were prescribed at the time of admission. They were admitted either through emergency room or from outpatient clinic. One child was randomly selected daily from the number of patients admitted in pediatric ward and intensive care unit during past 24 hours. These patients were followed during their hospitalization and relevant details of presenting symptoms and signs on admission, laboratory results, provisional diagnosis, antibiotics prescribed with dose and duration, change of antibiotics and its reason if mentioned, and final diagnosis were recorded in Performa. Since this was an observational study, no intervention was done. Prescribing of antibiotics and other drugs depended on the choice of consultant in charge of the admitted patient.

Data was analyzed by using SPSS (Statistical package for social sciences for window version 19). Frequencies were calculated for various antibiotics prescribed. 2 × 2 tables were used to look at the correlation between antibiotics used and antibiotic sensitivity of organisms isolated.

## 3. Results

A Total of 131 patients were included over 6 months of study period, in whom antibiotics were prescribed at the time of admission. The majority of these patients were between 1 and 5 years of age. Male to female ratio was 1 : 1. The mean duration of hospitalization was 2.7 days. Fever was the commonest symptom (84%) followed by diarrhea, vomiting and cough. Blood culture was done in 114 cases (87%), and the yield was positive only in 10 cases (8.8%). The commonest organism isolated from blood was *Salmonella* Typhi (18.2%). Ceftriaxone was found to be the most frequently prescribed antibiotic as an empirical therapy regardless of the admission diagnosis. Out of 131 patients 102 (7.86%) had received Ceftriaxone, followed by ampicillin. Children admitted with primary diagnosis of acute gastroenteritis 24/29 (83%) and UTRI 11/17 (65%), respectively, received Ceftriaxone as first line therapy (Table 1). Furthermore, out of 131 cases 114 were discharged on antibiotics, and the majority were on oral or injectable third generation cephalosporin irrespective of the admitting diagnosis. The third generation cephalosporin usage on discharge in conditions like acute gastroenteritis (82%), URTI (10%), and bronchiolitis (37.5%) was deemed irrational as these children can be discharge home without antibiotics once the initial blood culture was reported as sterile (Table 2).

## 4. Discussion

In our study, it was observed that Ceftriaxone was the most frequently prescribed first line antibiotic on admission and discharge, irrespective of diagnosis. The inappropriate and prolonged antimicrobial use favors the emergence of resistance organisms in hospitals. In our study, except in four cases of gastroenteritis in which significant leucocytes were found on stool examination, antibiotics should not have been prescribed. We could not find any justification for the use of antibiotics in these cases of gastroenteritis. As we had selected only those children, in whom antibiotics were prescribed at the time of admission, it is difficult to estimate the actual rate of antibiotics prescription in children admitted with gastroenteritis. Not only unnecessary prescription of antibiotics in acute gastroenteritis was observed, but it was also noticed that type of antibiotics prescribed was not appropriate in the majority of cases. For example in 78% cases, Ceftriaxone was prescribed as first line therapy on admission as well as being inappropriately prescribed at the time of discharge, despite a negative blood and stool culture results. We presume that in emergency department setup, patients presenting with acidosis and dehydration, younger age and physician's fear of “not treating bacterial infection” may be a major factors for overprescribing antibiotics in diarrhea and other acute viral illnesses. Another explanation is the lack of laboratory facilities in developing countries and perceived patient or parental pressure for prescribing antibiotics in the absence of bacterial infection. However, discontinuation of antibiotics after 48–72 hours in children showing clinical improvement and negative blood culture could be considered a step in the right direction.

Similarly, in acute respiratory tract infection and asthma, antibiotics are often not needed as viruses are the most common causes of respiratory tract infections. Even in those cases where antibiotics are needed, drug of choice is either penicillin or ampicillin. Use of Ceftriaxone for treatment of respiratory infection observed in our study was deemed inappropriate. Moreover, the use of antibiotics in 65% of children with URTI was higher than that of observed in Memphis, Tennessee, where 43% of children with uncomplicated URTI had received antibiotics [7]. In Kentucky Medicaid study, 60% patients with common cold had received antibiotics [8, 9]. In a recent survey of European primary care pediatrician, 43.5% of respondents overestimated the risks associated with not prescribing antibiotics [10].

In our study, pneumonia and bronchiolitis were the second commonest cause of hospitalization. This was also observed in other countries [8, 9]. Use of Macrolides and Quinolones in acute respiratory tract infections was not observed in our study as compared to other studies [11].

Antibiotics prescription in enteric fever, UTI, and meningitis was found to be rational in our study. Of the 25 patients with suspected enteric fever, 23 (92%) had received Ceftriaxone at the time of admission as a normal practice at AKUH due to prevalence of multidrug resistant organisms in Karachi and other parts of the world [12]. Multidrug resistant strains of Salmonella were seen in our study also. In a study from Mayo Clinic Children's Hospital, in Rochester, Minnesota, the most common reason for inappropriate antibiotic use was failure to discontinue or deescalate therapy [13]. Discontinuation of Ceftriaxone in 30% cases after negative blood culture indicates a rational approach in the use of antibiotics in our setup.

## 5. Conclusion

Our study demonstrates the inappropriate use of antimicrobials in acute febrile illnesses such as diarrhea and URTI irrespective of admission or discharge diagnosis. In addition, third generation cephalosporin was used in bronchiolitis and pneumonia despite the availability of first line therapy. There is a trend of continuing antibiotics on discharge and their use was neither substantiated by discharge diagnosis nor bacterial isolated on blood or other specimen cultures. There is a perceived patient or parental pressure for prescribing antibiotics in the absence of bacterial infection. Efforts are needed to educate physicians in rational use of antibiotics.

## Figures and Tables

**Table 1:** Diagnosis and antibiotics prescribed at the time of admission.

Serial number.	Diagnosis on admission	Total number of cases	Antibiotics prescribed on admission				
			Ceftriaxone *n* (%)	Ampicillin *n* (%)	Nalidixic acid *n* (%)	Ciprofloxacin *n* (%)	Multiple *n* (%)
1	Acute gastroenteritis	29	24 (83)	1 (3.5)	2 (7)	2 (7)	0
2	Enteric fever	25	23 (92)	2 (8)	0	0	0
3	Pneumonia	20	15 (75)	3 (15)	0	0	2 (10)
4	Upper respiratory tract infection	17	11 (65)	6 (35)	0	0	0
5	Urinarytract infection	10	8 (80)	0	1 (10)	1 (10)	0
6	Febrile seizure	8	6 (75)	2 (25)	0	0	0
7	Bronchiolitis	7	4 (57)	3 (43)	0	0	0
8	Meningitis	5	4 (80)	1 (20)	0	0	0
9	Reactive airway disease/asthma	2	0	2 (100)	0	0	0
10	Others	8	7 (87.5)	0	0	0	1 (12.5)

**Table 2:** Diagnosis and antibiotics prescribed at the time of discharge.

Serial number	Diagnosis on discharge	Total number of cases at discharge	Antibiotics prescribed on discharge						
			Cefixime *n* (%)	Ceftriaxone *n* (%)	Amoxicillin *n* (%)	Amox + Clav Acid *n* (%)	Nalidixic acid *n* (%)	Ciprofloxacin *n* (%)	Misc. *n* (%)
1	Acute gastroenteritis	22	15 (68.2)	3 (13.6)	—	—	2 (9.1)	2 (9.1)	—
2	Pneumonia	22	6 (27.3)	4 (18.2)	6 (27.2)	5 (22.7)	—	1 (4.6)	—
3	Enteric fever	22	10 (45.5)	10 (45.5)	1 (4.5)	—	—	—	1 (4.5)
4	URTI	10	1 (10)	—	7 (70)	2 (20)	—	—	—
5	Febrile seizures	8	3 (37.5)	3 (37.5)	2 (25)	—	—	—	—
6	Urinary tract infection	6	5 (83.3)	—	—	—	—	1 (16.7)	—
7	Bronchiolitis	8	3 (37.5)	—	3 (37.5)	1 (12.5)	—	—	1 (12.5)
8	Meningitis	4	—	4 (100)	—	—	—	—	—
9	RAD/Asthma	2	—	—	—	2 (100)	—	—	—
10	Others	10	4 (40)	3 (20)	2 (20)	—	—	—	1 (10)

## References

[1] Cosgrove SE, Carmeli Y (2003). The impact of antimicrobial resistance on health and economic outcomes. *Clinical Infectious Diseases*.

[2] Saied T, Elkholy A, Hafez SF (2011). Antimicrobial resistance in pathogens causing nosocomial bloodstream infections in university hospitals in Egypt. *American Journal of Infection Control*.

[3] Trostle J (1996). Inappropriate distribution of medicines by professionals in developing countries. *Social Science and Medicine*.

[4] Nizami SQ, Khan IA, Bhutta ZA (1995). Differences in self-reported and observed prescribing practice of general practitioners and paediatricians for acute watery diarrhoea in children of Karachi, Pakistan. *Journal of Diarrhoeal Diseases Research*.

[5] Nizami SQ, Khan IA, Bhutta ZA (1996). Self-reported concepts about oral rehydration solution, drug prescribing and reasons for prescribing antidiarrhoeals for acute watery diarrhoea in children. *Tropical Doctor*.

[6] Nizami SQ, Bhutta ZA, Molla AM (1996). Efficacy of traditional rice-lentil-yogurt diet, lactose free milk protein-based formula and soy protein formula in management of secondary lactose intolerance with acute childhood diarrhoea. *Journal of Tropical Pediatrics*.

[7] Arnold KE, Leggiadro RJ, Breiman RF (1996). Risk factors for carriage of drug-resistant Streptococcus pneumoniae among children in Memphis, Tennessee. *Journal of Pediatrics*.

[8] Mainous AG, Hueston WJ, Clark JR (1996). Antibiotics and upper respiratory infection: do some folks think there is a cure for the common cold?. *Journal of Family Practice*.

[9] Mainous AG, Hueston WJ (1998). The cost of antibiotics in treating upper respiratory tract infections in a medicaid population. *Archives of Family Medicine*.

[10] Grossman Z, del Torso S, Hadjipanayis A, van Esso D, Drabik A, Sharland M (2012). Antibiotic prescribing for upper respiratory infections: European primary paediatricians’ knowledge, attitudes and practice. *Acta Paediatrica*.

[11] Huilan S, Zhen LG, Mathan MM (1991). Etiology of acute diarrhoea among children in developing countries: a multicentre study in five countries. *Bulletin of the World Health Organization*.

[12] Bhutta ZA (1996). Therapeutic aspects of typhoidal salmonellosis in childhood: the Karachi experience. *Annals of Tropical Paediatrics*.

[13] Levy ER, Swami S, Dubois SG, Wendt R, Banerjee R (2012). Rates and appropriateness of antimicrobial prescribing at an academic children’s hospital, 2007–2010. *Infection Control and Hospital Epidemiology*.

